# Supplementary material for: Cross-Sectional Study Assessing Management Practices and Udder Health in California Sheep Flocks and Seroprevalence of Small Ruminant Lentivirus
Source: Animals (Basel). 2024 Aug 13;14(16):2332. doi: 10.3390/ani14162332 (PMC11350894; doi:10.3390/ani14162332)
Supplement: Supplementary file 1 [file animals-14-02332-s001.zip › animals-3123374-supplementary.pdf]

UNIVERSITY OF CALIFORNIA
Agriculture and Natural Resources | Cooperative Extension

May 2022

California Ewe Mastitis and Lamb Survivability

Rose Digianantonio, DVM, MPH, Livestock Reproduction and Herd Health Resident, UC Davis,
Roselle Busch, DVM, Sheep and Goat Extension Veterinarian, UC Davis

Has the lambing season left you sick of grafting orphaned lambs and/or dealing with bottle babies? It may be necessary to improve ewe udder health on your farm.

To highlight areas for future research, Dr. Roselle Busch, Sheep and Goat Extension Veterinarian at UC Davis, and her research lab in collaboration with UCCE Livestock Advisors have developed a survey for sheep producers about management of ewes and care of mastitis. If you own or manage breeding ewes and are willing to participate, please scan the QR code below or click here and complete the survey. This survey will take less than 10 minutes and can help us improve ewe and lamb health

What is Mastitis and why do we want to prevent it?

In pasture-raised sheep operations, the highest percentage of lamb losses occur within the first 72 hours of birth. This directly impacts productivity and profitability of the flock. Udder pain is one of the main reasons for ewes to reject lambs. In range or pasture rearing systems, rejection of the lamb by the ewe can create orphan lambs or lead to lamb starvation if noticed too late.

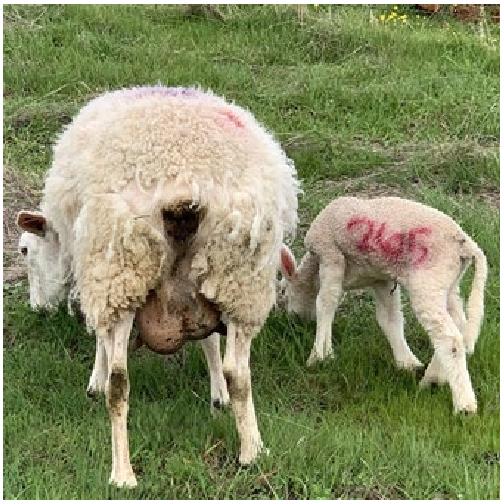

A ewe that can't raise a twin lamb and has an asymetrical udder and wool break should be checked for mastitis.
Source: Dan Macon/Roger Ingram

Mastitis is inflammation of the mammary gland/udder. The three types of mastitis that we typically think about and their descriptions are below

1. Clinical mastitis: Visible signs of milk and/or udder abnormalities.

2. Sub-clinical mastitis: Elevated inflammatory cells in milk, decreased milk production, but no obvious abnormalities in milk appearance or udder appearance.

3. OPP (Ovine Progressive Pneumonia Virus): "Hard Bag." Typically, the udder is firm, but non-painful and has little to no milk production. OPP is contagious between sheep.

Ewes are at increased risk for mastitis due to different environmental and individual factors. A few include poor nutrition, unsanitary housing conditions, litter size, and poor teat conformation. No matter the cause, any of these forms of mastitis lead to production losses on the farm. Mastitis prevention and control may limit lamb production losses, the number of bottle lambs, and improve lifetime productivity of your ewes. Help us improve our knowledge in this area by taking our survey.

Please contact Dr. Roselle Busch with any questions:
rcbusch@ucdavis.edu | (530)574-8208
